# ChatGPT and other artificial intelligence chatbots and biomedical writing

**DOI:** 10.20517/jca.2023.13

**Published:** 2023-03-31

**Authors:** Priyatansh Gurha, Nadeem Ishaq, Ali J. Marian

**Affiliations:** 1 Center for Cardiovascular Genetics, Institute of Molecular Medicine, The University of Texas Health Science Center, Houston, TX 77030, USA.; 2 Octia Corporation, Houston, TX 77027, USA.

**Keywords:** Artificial intelligence, machine learning, language learning models, chatbot

We asked ChatGPT whether it should be listed as an author of the text that it generates in response to questions. Here is how it responded:

“Hello! As an AI language model, I don’t have a personal identity or consciousness in the traditional sense, and I don’t create content on my own. I was created by OpenAI, a research organization, and trained on vast amounts of text from the internet to generate human-like language in response to user prompts.

However, the content that I generate is still subject to copyright laws, and the ownership of the content that I generate depends on the specific context and use case. In general, if someone uses my responses in a creative work, they would likely be considered the author of that work, but the original content generated by me would still be subject to copyright laws and owned by OpenAI.”

Well said, ChatGPT!

An artificial intelligent (AI) chatbot, such as ChatGPT or an AI writer, cannot be an author and must not be listed as an author in a scientific publication in *The Journal of Cardiovascular Aging* (*JCA*). The main reason for this declaration is our conviction that an author is conveying his/her nuanced interpretation of the data and hence, insight beyond simply synthesizing the sentences based on the pre-existing texts on the internet.

It is also important to recognize that the (human) authors cannot claim ownership of the text generated by ChatGPT and similar chatbots. Using such data without proper credit and without extensive edits is a red flag for plagiarism. The authors who choose to use chatbots to generate sentences, paragraphs, or the whole document must avoid plagiarism and acknowledge the use of such programs in their writings. The irrelevance of this policy to the original research articles is self-evident, as the applications of the AI-based programs, such as ChatGPT, to this category of articles are currently limited, if not nil. The assertion mainly pertains to the review articles, commentaries, perspectives, or a similar category of articles. In such format articles, a chatbot has the potential to generate relevant texts. The current chatbots, however, are rather elementary in generating in-depth and critical scientific writings that could serve as insightful articles. The programs use algorithms, referred to as language learning models (LLMs), to search an extremely large number of texts on the internet to predict sentences in response to queries. With the advances in technologies, however, it is not unreasonable to expect that in the future, a chatbot to generate a review, commentary, or perspective article that resembles, matches, or even supersedes an article written by “an authentic author”, namely an expert human scientist. Nevertheless, the essence of writing a good review article, a commentary, or a perspective is to offer one’s insight and, to a feasible extent, new insight into a scientific topic. A scientist who is an expert in a field, by looking at the same set of data that are available to the non-experts, can traverse beyond the data to extract new insight and convey his/her message. That is essential for a good review article. Consequently, a chatbot lacking insight cannot be an author.

The large language models, as they improve, are expected to find useful utilities in scientific writing. However, one must be aware of the limitations of these programs and consider texts generated by the chatbots provisional requiring careful evaluation for validity and editing of incorrect statements^[1]^. This is particularly important in scientific writing as chatbots lack understanding. Those who utilize *in silico* algorithms to identify new biological events, such as protein-protein interactions, based on text-mining, fully recognize the limitations of the findings, which are provisional at best and require validation through actual experimentation.

In addition to scientific writings, chatbots and other LLMs have several biomedical and clinical applications, such as facilitating clinical diagnosis and treatment, and analyzing large datasets to discover patterns, for example, for drug targets, drafting medical letters, teaching patients and trainees, and editing writing for typos and grammar errors, which we are not discussed. One challenge, of course, is the input data used to extract information. With the publication of approximately 2 million biomedical articles per year, including very low-quality articles, in about 30,000 biomedical journals, bias in data collection, interpretation, and reporting, and prevalent scientific misconduct, the LLM-based programs will face considerable risk of generating erroneous outputs. The daunting challenge requires critical and insightful analysis of the data by expert minds to discern reliable data, which is not simply possible by text mining. In clinical medicine, the preliminary studies suggest that ChatGPT could generate reasonably but not perfectly accurate responses to medical questions, write patient clinic letters, or summarize discharge notes^[2–6]^. However, all ChatGPT-generated medical documents must be reviewed and verified by experts.

As the programs technically become more advanced, they are expected to become more popular and gain widespread use. The increasing demand would usher in the commercialization of the products and subscription-based access, which is already in place for certain chatbots. We hope that the commercialization will not restrict access of the scientists to the platforms that could synthesize and extract advanced information from the existing data.

The editors of *JCA* are attuned to identifying writing that lacks nuanced insight and fails to advocate a new understanding. Such articles are not expected to receive sufficient priority for publication in *JCA*.
